# Maladie de Verneuil: un véritable défi thérapeutique

**DOI:** 10.11604/pamj.2014.17.316.3698

**Published:** 2014-04-25

**Authors:** Maha Mael-Ainin, Karima Senouci

**Affiliations:** 1 Service de Dermatologie, CHU Ibn Sina, Université Mohamed V, Souissi, Rabat, Maroc

**Keywords:** Maladie de Verneuil, abcès, affection inflammatoire, Verneuil's disease, abscess, inflammatory disease

## Image en medicine

La maladie de Verneuil est une affection inflammatoire chronique du follicule pileux, caractérisée par des poussées itératives de nodules douloureux, d'abcès, de fistules drainantes et de cicatrices hypertrophiques en cordes. Les lésions siègent essentiellement aux niveaux des régions anatomiques riches en glandes sudoripares apocrines: régions inguinales, génitales, périanales, axillaires, inter ou sous mammaires. Des localisations atypiques sont possibles surtout chez l'homme, notamment au niveau de la nuque, thorax et abdomen. Les tableaux cliniques sont multiples: formes intermittentes ou continues mineures et majeures. La qualité de vie des patients est généralement altérée. Le traitement est médico-chirurgical reposant sur une antibiothérapie prolongée, à large spectre, parfois sur la corticothérapie, le zinc, les sulfones ou l'Acitrétine. Selon le stade évolutif, des excisions limitées ou larges peuvent être associées. Devant les rechutes fréquentes, des alternatives thérapeutiques sont en cours d’évaluation notamment les agents anti-TNF, les lasers CO2 ou Nd Yag et les injections de toxines botuliques. Mr N.L, âgé de 28 ans, sans antécédents pathologiques particuliers, qui a présenté depuis l’âge de 19ans des nodules douloureux, des abcès et des fistules siégeant au niveau des aisselles, des plis inguinaux, de la nuque et du thorax. Les lésions évoluait par poussées subintrantes avec depuis deux ans une augmentation du nombre des lésions, de la durée des suppurations et apparition de cicatrices hypertrophiques au niveau de la nuque et du thorax. Diverses thérapeutiques ont été utilisées notamment l'Amoxicilline protégée, les macrolides, le Metronidazole, les cyclines et les rétinoïdes ainsi que des exérèses locales. Devant la persistance de la symptomatologie, le patient a été mis sous Disulone à la dose de 2mg/kg/jour.

**Figure 1:**
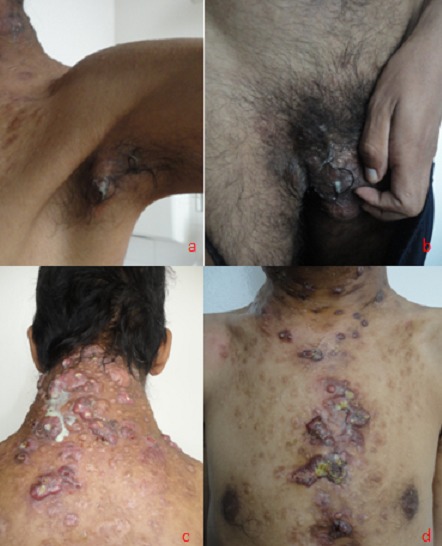
(A): Abcès fistulisé au niveau axillaire gauche; (B): Abcès fistulisé au niveau inguinal droit; (C): Cicatrices chéloïdes suppurées de la nuque et du dos; (D): Cicatrices chéloïdes surmontées de croûtes mélicériques au niveau du thorax

